# Bias in phylogenetic tree reconciliation methods: implications for vertebrate genome evolution

**DOI:** 10.1186/gb-2007-8-7-r141

**Published:** 2007-07-16

**Authors:** Matthew W Hahn

**Affiliations:** 1 Department of Biology and School of Informatics, E. 3rd Street, Indiana University, Bloomington, IN 47405, USA

## Abstract

**Background:**

Comparative genomic studies are revealing frequent gains and losses of whole genes via duplication and pseudogenization. One commonly used method for inferring the number and timing of gene gains and losses reconciles the gene tree for each gene family with the species tree of the taxa considered. Recent studies using this approach have found a large number of ancient duplications and recent losses among vertebrate genomes.

**Results:**

I show that tree reconciliation methods are biased when the inferred gene tree is not correct. This bias places duplicates towards the root of the tree and losses towards the tips of the tree. I demonstrate that this bias is present when tree reconciliation is conducted on both multiple mammal and *Drosophila *genomes, and that lower bootstrap cut-off values on gene trees lead to more extreme bias. I also suggest a method for dealing with reconciliation bias, although this method only corrects for the number of gene gains on some branches of the species tree.

**Conclusion:**

Based on the results presented, it is likely that most tree reconciliation analyses show biases, unless the gene trees used are exceptionally well-resolved and well-supported. These results cast doubt upon previous conclusions that vertebrate genome history has been marked by many ancient duplications and many recent gene losses.

## Background

Comparative genome sequencing of many closely related organisms has revealed remarkable similarities in the total number of genes among taxa. However, this similarity in total number masks numerous changes in the underlying identity of the genes in each species (for example, [1-4]). Differences in the identities of constituent proteins arise because genes are gained and lost throughout evolution: gains occur through the duplication of whole genomes or individual genes, and losses occur via the deletion or pseudogenization of previously functional genes. The importance of gene duplication has been appreciated for a long time [5,6], while the importance of gene loss has only recently attracted attention [7,8].

Though there exist many widely used methods for studying the evolution of nucleotide substitutions, the study of gene gain and loss presents many more challenges. These challenges exist in both data collection (for example, accurate assembly of whole-genome shotgun sequencing) and data analysis (for example, accurate estimation of duplication times). Fortunately, a number of complementary methods have arisen to enable researchers to accurately study gene gain and loss at a genome-wide level. The most commonly used methods compare the species tree that describes the relationships among taxa to the gene tree inferred from the sequences of the gene family being studied [9-14]. By reconciling the gene tree with the species tree, both gene gains and losses can be inferred and mapped onto the species tree. This method has been applied to many individual gene trees (for example, [15]), and is only now finding wider usage in whole genome analyses (for example, [16]).

A major problem with the gene tree/species tree reconciliation method is that it assumes that both trees are free from error [9]. While thousands of orthologous genes can be used to construct the species tree - with commensurately increased confidence in any topology - each individual gene tree can only be inferred from the gene family it represents. For this reason, some methods for carrying out the reconciliation explicitly take into account the support at every node, usually via bootstrap values (for example, [13,14]). Nodes with little support are collapsed, which prevents the non-parsimonious addition of both duplications and deletions. However, because of the limited number of characters used to build each gene tree and the vagaries of reconstruction methods, there may be incorrectly inferred topologies even with 100% bootstrap support (for example, [17,18]).

In this paper I describe a consistent bias in such tree reconciliation methods. This bias leads to an overestimation in the number of duplicates placed near the root of the species tree, and an overestimation of the number of losses across the tree. The bias increases when topologies with weaker support are allowed, though it appears to exist even when poorly supported topologies are taken into account. Finally, I show how this bias has led to incorrect inferences regarding the nature of vertebrate genome evolution, but how careful analysis of the data can still allow some conclusions to be made.

## Results and discussion

### Tree reconciliation bias

**Figure 1:**
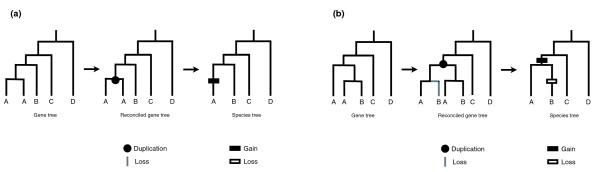
Two examples of tree reconciliation. In both **(a) **and **(b) **the leftmost tree represents the gene tree, the middle tree the reconciled gene tree showing the duplications and losses, and the rightmost tree shows the species tree with gains (duplications) and losses mapped onto the appropriate branches. The reconciled gene trees represent what the gene tree would look like including lost genes (grey branches).

Tree reconciliation proceeds by adding the minimum number of gains and losses to the species tree to make it consistent with the gene tree. Figure 1 gives two examples of such reconciliations, explicitly showing the inferred history of gain and loss and how these are then mapped onto the species tree. If both the species tree and the gene tree are correct, then the various reconciliation algorithms in use should all recover the correct history of duplication and loss, albeit with varying computational efficiency [11]. These methods also assume that there are no missing data, a problem that could result in incorrectly inferred gene losses [14].

**Figure 2:**
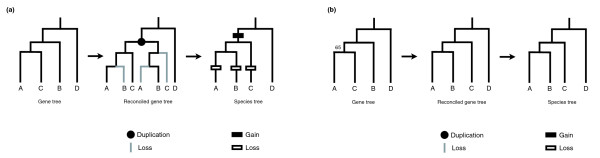
Tree reconciliation bias. **(a) **The effect of wrongly inferring the gene tree: the addition of one duplication and three losses. **(b) **An example where a low bootstrap value (65%) below the cut-off results in the collapse of the gene tree. As a result, no duplications or losses are inferred.

If one of the trees is not correct (I assume in the following that this will usually be the gene tree), then additional gains and losses are added to the species tree in order to completely reconcile the two trees. Figure 2a gives an example of an incorrectly inferred gene tree, one that simply has the branching order of two of the homologous genes switched (lineages B and C). In order to reconcile this gene tree with the species tree, a single duplication must be placed above the point at which the affected lineages split and three separate gene losses must occur on the terminal lineages (Figure 2a). When tree reconciliation methods are conducted taking into account bootstrap (or other) support for each node, incorrectly inferred topologies may be collapsed back to the branching order in the species tree. This has the effect of minimizing the number of proposed gains and losses of genes. Figure 2b shows the same example as in Figure 2a, but relatively low bootstrap support (65%) has been given to the node that will cause the extra duplication and deletions. In this case, any bootstrap cut-off used that is above 65% will result in the collapse of this node, and no duplications or losses would be inferred.

As discussed above, the small number of characters used to build a given gene tree means that many trees may be incorrectly inferred. Even with relatively long sequences, the requirement that trees are found for every family in a genome in reasonable time means that approximate methods, such as neighbor-joining [19] must be used. If bootstrap support at every node is needed, likelihood-based methods for inferring gene trees become computationally prohibitive even for a small number of trees. It is also recognized that high bootstrap support can be dependent on the exact phylogenetic method and model of sequence evolution used [17,18,20-22]. Furthermore, short inter-node distances can result in individual gene trees that are different from the species tree because of incomplete lineage-sorting, and not because of any errors in tree reconstruction methods [23-25]. For all of these reasons, it appears likely that many of the gene trees in whole genome studies will have been incorrectly inferred, or will have artificially high bootstrap support for incorrect topologies. Additionally, some of the most widely used methods for conducting reconciliation do not make it possible to consider the support for topologies [10,11], so that no allowance can be made for incorrect topologies.

Errors in gene tree reconstruction result in two consistent biases in tree reconciliation: more duplications must be assigned to branches further up the tree, towards the root; and more losses must be assigned to branches below these duplications. As shown in Figure 2a, topological disagreements between the species tree and gene tree always result in the placement of duplications above the branches that are inconsistent between the two trees; this is the only way to reconcile the differences. As a result of the duplications added to the tree, multiple losses must also be added, always on lineages further toward the tips. This bias results in an inferred history of many older gene gains and many recent gene losses.

### Accounting for the bias

One further characteristic of the additional duplications that are added due to errors in tree topologies is that they will only be assigned to branches with more than two descendant lineages. This effect occurs because sub-topologies of a larger tree involving two or fewer lineages cannot be incorrectly inferred (for example, the topology [A, B] is the same as [B, A]). Inconsistencies between the gene and species tree must be due to the mis-ordering of three or more branches (for example, the topology [A, B]C] is not the same as [A, C]B]). Tree reconciliation can only proceed by adding duplications to lineages preceding mis-ordered branches (Figure 2a), and, therefore, can only be added to lineages with three or more descendants.

The effect of this bias means that terminal lineages and many of the lineages leading to them will not be incorrectly assigned duplications. Terminal lineages ('tips') and lineages giving rise to only two terminal lineages ('doublets'; for example, the branch leading to [A, B] in the example species tree) will not have duplications added to them erroneously, no matter how inconsistent the gene tree and species tree are with each other. Therefore, information about the number of gene duplicates inferred on these branches should be accurate. I consequently define these branches of the species tree as 'informative,' and use them in further comparisons below.

A further possibility to account for reconciliation bias on non-informative branches is to iteratively remove descendant branches from the gene trees to be reconciled. Because incorrect duplications are placed on these branches only when there are genes from three or more descendant branches, pruning the trees so that only two or fewer lineages are represented may allow for more accurate reconstruction of the number of duplicates on these branches. This method then essentially turns 'non-informative' branches into 'informative' branches by reducing the possibility that gene trees are incorrect. Further work will need to be done as to how exactly such pruning is implemented.

Unfortunately, estimates of the number of losses appear to be biased across all lineages. Because duplications can be incorrectly placed as deep as the branch leading to the root - and no losses can be inferred on this branch - all branches of the species tree descend from lineages that could contain false duplications. This means that the number of gene losses will be over-estimated for all branches of the tree, and will increase in number towards the tips.

### Molecular evidence for reconciliation bias

In order to provide an example of the bias described here, I conducted tree reconciliation for 9,920 gene trees from 6 mammalian genomes and 11,388 gene trees from 12 *Drosophila *genomes (Materials and methods). To show the effect that increasing errors have on the number of inferred gains and losses, I carried out the reconciliations with six different values for the bootstrap cut-off: 100%, 90%, 80%, 70%, 60%, and 50%. My prediction is that the number of duplications on non-informative branches should increase as the bootstrap cut-off decreases. This is because more topologies with lower support (which are likely to be incorrect topologies) are included with lower cut-offs. There should be no directional effect on the number of duplications inferred on informative branches. In addition, the number of losses should increase across all branches as the bootstrap cut-off decreases.

**Figure 3:**
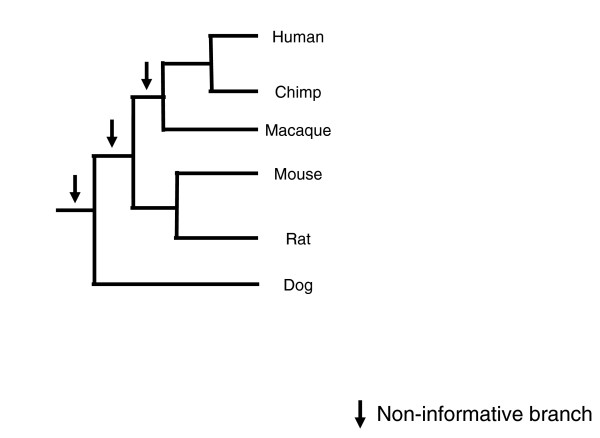
Mammalian species tree. A phylogenetic tree of the six species considered in the text is shown (branches are not proportional to time). Non-informative branches are marked with an arrow.

**Figure 4:**
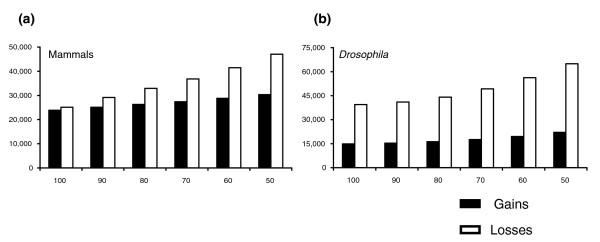
The effect of tree reconciliation bias. The graphs show the relationship between the number of gains and losses inferred as a function of the bootstrap cut-off used for **(a) **the mammalian tree, and **(b) **the *Drosophila *tree. The numbers represent the sum of gains and losses across all branches of the species trees.

Figure 3 shows the mammalian species tree and Figure 4a the number of gains and losses inferred across the tree at varying bootstrap cut-offs. This tree contains three non-informative branches (indicated by arrows) and eight informative branches. The number of duplications on non-informative branches does in fact increase with decreasing bootstrap cut-offs; this trend is strongly significant (Table 1). Summing across all non-informative branches, we would infer 14,966 duplications with a 100% bootstrap cut-off, but 22,031 with a 50% cut-off. Also as predicted, the number of losses on all branches increases with decreasing cut-offs: the total number increases from 25,092 to 47,074 as one goes from a 100% to a 50% bootstrap cut-off. On average, a 10% decrease in the bootstrap cut-off used results in a 16% increase in the number of inferred losses on any given branch and an 8% increase in the number of inferred gains on non-informative branches. The same trends are found for the *Drosophila *tree, with significant increases in duplication and loss resulting from decreasing support for tree topologies (Figure 4b and Table 1).

**Table 1:** Correlation between bootstrap cut-off and numbers of inferred gains and losses

	Mammals	*Drosophila*
**All branches**		
Duplications	-0.99*	-0.96*
Losses	-0.99*	-0.96*
**Non-informative branches**		
Duplications	-0.99*	-0.97*
Losses	-0.99*	-0.95*
**Informative branches**		
Duplications	0.99*	0.97*
Losses	-0.99*	-0.97*
**Doublet branches**		
Duplications	-0.82^†^	-0.12
**Tip branches**		
Duplications	0.99*	0.97*

*P < 0.00001, ^†^*P *< 0.05.

**Figure 5:**
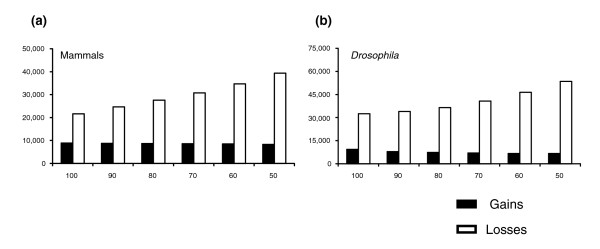
Accounting for tree reconciliation bias. The graphs show the relationship between the number of gains and losses inferred as a function of the bootstrap cut-off used for **(a) **the mammalian tree, and **(b) **the *Drosophila *tree. The numbers represent the sum of gains and losses across only informative branches of the species trees.

One surprising result is that there appears to be a slight but significant correlation between the number of gains on informative branches and the bootstrap cut-off used - the number of duplications increases with increasing bootstrap cut-off values. This trend is the opposite of the one predicted for non-informative branches, but is significant for both mammals and *Drosophila *(Figure 5 and Table 1). In comparison to the effect bootstrap cut-off values have on non-informative branches, the consequence of this pattern is much smaller. The total number of inferred duplications on informative mammalian branches only falls from 8,870 to 8,332 going from a 100% to a 50% bootstrap cut-off. This equates to an average of 1.3% duplications removed for every 10% decrease in the bootstrap cut-off (the value for *Drosophila *is a 3.4% decrease for every 10%).

**Figure 6:**
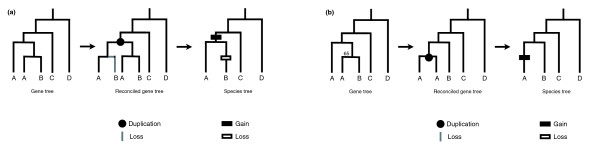
Slight bias towards placing duplicates on the tips of the tree. **(a) **Shows how gains and losses would be inferred for the gene tree shown. **(b) **Taking into account bootstrap support can result in placing duplicates towards the tips as gene tree topologies are collapsed.

The apparent cause of this slight bias is shown in Figure 6. As the bootstrap cut-off is increased, even relatively well-supported topologies will be collapsed. The aim of collapsing nodes is to minimize the total number of gains and losses that must be invoked to explain the history of any given gene tree. The minimum number of changes can be achieved by pushing all duplicates towards the tips of the tree, as no further losses can be added. The addition of duplicates to non-informative branches always results in an equal or greater number of losses on descendant lineages, and, therefore, a greatly increasing total number of changes. This slight bias has consequences for methods that attempt to choose the 'true' gene tree by minimizing gains and losses (for example, [26,27]): placing duplicates towards the tips of the tree will often be favored. The pattern shown in Figure 6 may also be caused by missing data, such that the gene that has been 'lost' (one of the B genes) results in an inferred duplication.

The above explanation for the positive relationship between bootstrap cut-offs and number of duplications predicts that the increase seen on informative branches should be found predominantly on tip branches; placing duplications on the few informative branches that lead to two descendant lineages does not minimize the total number of changes. In fact, this is exactly what is observed in both mammals and *Drosophila*. As shown in Table 1, there is no significant correlation between the number of gains and bootstrap cut-off for doublet branches in *Drosophila *(*r *= -0.12, *P *= 0.83), and only a marginally significant relationship in mammals, but in the opposite direction from the relationships found earlier (*r *= -0.82, *P *= 0.045). The correlation on just tip branches remains strong and highly significant (mammals: *r *= 0.99, *P *= 0.0001; *Drosophila*: *r *= 0.97, *P *= 0.001).

### Independent estimation of gene gain and loss

As a further check on the accuracy of the number of estimated gene duplicates on informative branches, I estimated the number of gene duplicates and gene losses using an unrelated likelihood method [3]. This method does not use gene trees, and is therefore expected to provide independent support for the inferred number of duplications on informative branches. Briefly, the method infers gains and losses only from the number of copies of genes present in each of the species included, and does not consider the relationships among the constituent genes. I do not expect there to be any similarity between the numbers of losses estimated by the two methods, on any branches of the species tree.

**Figure 7:**
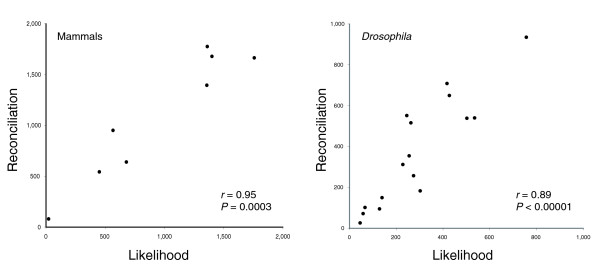
Relationship between tree reconciliation and likelihood methods for estimating the number of gene gains. The number of gene duplicates inferred on only informative branches of the **(a) **mammalian tree, and **(b) ***Drosophila *tree are shown.

Figure 7 shows the correlation in the number of duplications inferred across informative branches by the two methods for both mammals and *Drosophila*. There are highly significant correlations in both: *r *= 0.95 (*P *= 0.0003) for mammals and *r *= 0.89, (*P *< 0.00001) for *Drosophila*. This provides evidence for the accuracy of tree reconciliation methods when considering only the number of genes gained on informative branches (those with two or fewer descendants). Duplications on these branches should be correctly inferred by all methods. Including non-informative branches, however, the correlation in number of gene duplications inferred between methods is no longer significant (mammals: *r *= 0.25, *P *= 0.48; *Drosophila*: *r *= -0.18, *P *= 0.43). As an example of the disconnect between the two methods when applied to non-informative branches, the likelihood method infers 15 gene duplications on the short (approximately 4 million year) branch leading to the 4 non-canine mammals; tree reconciliation infers the gain of 2,774 genes on the same branch.

The number of gene losses also appears to be badly estimated by tree reconciliation methods: correlation with likelihood estimates is either non-significant (mammals: *r *= 0.52, *P *= 0.18) or mildly significant (*Drosophila*: *r *= 0.63, *P *= 0.01). Even the mildly significant correlation observed for losses is deceptive - the number of losses estimated by the reconciliation method is, on average, seven times as great as the number estimated by likelihood. For example, on the lineage leading to *Drosophila melanogaster *the likelihood method infers the loss of 547 genes since the split with *D. simulans *(approximately 5 million years ago [28]). The tree reconciliation method infers the loss of 3,461 genes.

On average, the number of duplicates on informative branches inferred via tree reconciliation is 1.25 (*Drosophila*) to 1.5 (mammals) times as high as the number inferred via the likelihood method (Figure 7). The higher estimate using tree reconciliation may have two causes: the slight bias towards placing duplicates on the tips of the tree with increasing bootstrap cut-off stringency; or the tendency for the likelihood method to undercount the number of gains and losses when both types of events occur in the same gene family on the same branch of the phylogenetic tree [3]. However, the discrepancy between the two methods remains the same on informative branches even when using a bootstrap cut-off of 60%, suggesting that the more likely cause is underestimation via the likelihood method.

### Implications for vertebrate genome evolution

The bias described here will affect all previous studies that have used tree reconciliation methods. The effects of this bias will be mitigated by using reconciliation methods that take into account bootstrap support (for example, [13,14]) rather than those that do not [10,11]; the effects will be further minimized by using more accurate gene tree inference methods (such as maximum likelihood) rather than fast and approximate methods (such as neighbor-joining). Finally, as the vagaries of tree inference are strongly influenced by the particular information contained within the protein sequences of the genes being considered, reconciliation of any particular gene tree may or may not be affected by the bias described here. However, when genome-scale analyses are conducted, even the slight effects of reconciliation bias will be magnified across the thousands of gene trees considered.

In a recent paper, Blomme *et al*. [16] used tree reconciliation to infer the history of gene gain and loss among seven vertebrate species. Gene trees for 8,165 families were constructed using neighbor-joining and reconciled with the known species tree using a 70% bootstrap cut-off [16]. Two of the conclusions of the paper were that "the majority of duplicated genes in extant vertebrate genomes are ancient," and that "all vertebrates continue to lose duplicates that were created at much earlier times." Based on the biases in tree reconciliation methods demonstrated here, it appears likely that the patterns observed by Blomme and colleagues are largely artifactual. The same biases that tree reconciliation methods show - spurious inferences of a large number of ancient duplications followed by an even larger number of recent losses - are precisely the results of their analyses. Given the relatively low bootstrap cut-offs used in the published analyses, one would expect a reduction in both gains and losses with increasing topological stringency.

One further conclusion of the Blomme *et al*. study relates to the association of a large number of inferred duplications with multiple whole genome duplications (WGDs). It is not immediately obvious that the precise placement of gene duplications on the non-informative branches of the vertebrate tree should be affected by reconciliation bias, and, therefore, that the timing of WGD events should be wrongly inferred. There is no significant correlation between the number of duplications inferred on a branch and the distance from the tips, though there is a trend in that direction (*Drosophila*: *r *= 0.44, *P *= 0.39). This indicates that there does not appear to be a bias (among non-informative branches) in placing duplicates on the root branch, exactly where two WGD events are inferred in vertebrate history.

However, there is one interesting possibility for a specific bias in the placement of gene duplications: if topological discordance between the gene tree and species tree is due to incomplete lineage-sorting, then a large number of duplications from many different gene trees will be placed on the branch immediately preceding such an event. Incomplete lineage-sorting is due to short inter-node distances, such that polymorphism in the ancestral population is not completely fixed between speciation events. Incomplete lineage-sorting can result in disagreements between gene trees and species trees, even though none of the inferred gene trees is incorrect *per se *[23,24]. These disagreements can extend to whole genome analyses of single-copy orthologs, where no single gene tree is found from the majority of orthologs considered (for example, [25]).

As there appears to be an instance of incomplete lineage-sorting among the *Drosophila *[25], I asked whether a large number of duplications were placed on the branch preceding the topological discordance (the branch marked with an asterisk in Additional data file 1). As predicted, a large number of duplications were inferred on this branch: 2,757 in the best-supported topology, compared to 278 and 415 duplications on the non-informative branches above and below this one. The number of duplications inferred on the branch preceding the incomplete lineage-sorting was much higher in the two alternative topologies as well (data not shown). These analyses appear to show that short divergence times between speciation events can lead to an excess of inferred duplication events. One case in which incomplete lineage-sorting is common is during adaptive radiations - such radiations are notoriously hard to construct consistent species trees for [23]. This implies that tree reconciliation analyses will associate a large number of duplication events with adaptive radiations. Methods that allow for non-binary species trees (for example, [14,29]) should be used in these cases so that a large number of incorrect duplications are not inferred. Though it does not seem that there has been an adaptive radiation at the origin of the vertebrate species considered by Blomme *et al*. [16], caution should be used in inferring WGD events from the large number of duplications placed on any particular branch by tree reconciliation methods.

## Conclusion

The sequencing of a large number of whole genomes has made it possible to study patterns of gene gain and loss on an enormous scale. Even though methods for inferring gain and loss have been around for almost 30 years, only with the analysis of whole genomes has the effect of small biases become clear. The net effect of the bias in tree reconciliation methods demonstrated here is that the number of gene losses inferred should not be taken at face value, and the number of duplications inferred should be parsed with care.

How might we overcome the bias in current reconciliation methods? Algorithms that provide an estimate of statistical support for each inferred gain or loss (for example, [12,30]) or that take into account the length of species tree branches may both offer improvements to current methods. This latter possibility offers a way to improve inferences because short species tree branches are the ones that are most likely to lead to wrongly inferred gene trees, as is seen in the case of incomplete lineage sorting. It is also important to note that most of the biases described here occur when there are equal numbers of genes among taxa - if there are unequal numbers of genes, then duplications and losses can be inferred from presence/absence information. In this case the problem simply reduces to one concerning the evolution of copy number, which is exactly the approach of the likelihood method mentioned above. But this method is also not without its own biases [3].

Finally, biases in tree reconciliation methods cast doubt on previous work into the evolution of vertebrate genomes. However, the results presented here cannot disprove previous results, as an excess of ancient duplicates and recent gene losses were inferred even when using bootstrap cut-offs of 100%. Discovering the true pattern of vertebrate genome evolution will require simulation results, better gene trees, more data, or some combination of all three.

## Materials and methods

### Gene family data

Mammalian gene families were taken from Ensembl version 41 [31] for human (*Homo sapiens*), chimpanzee (*Pan troglodytes*), rhesus macaque (*Macaca mulatta*), mouse (*Mus musculus*), rat (*Rattus norvegicus*), and dog (*Canis familiaris*). I included only the longest isoform of each gene in the analysis. The resulting dataset includes 119,746 genes in 9,990 gene families across all six species. *Drosophila *gene family data are from the 12-genomes consortium [32]. This dataset includes 149,097 genes in 11,521 gene families across the 12 species. See Hahn, Han, and Han (in review) and Hahn, Demuth, and Han (in review) for more details on both datasets.

### Gene trees

Amino acid alignments for each mammalian gene family were downloaded from Ensembl. Alignments for the *Drosophila *proteins were made using MUSCLE [33]. Neighbor-joining trees for both sets of families were generated in PHYLIP [34] using JTT protein distances and 100 bootstrap runs. Gene trees could be constructed for 9,920 of the 9,990 mammalian gene families and 11,388 of 11,521 *Drosophila *gene families (PHYLIP could not handle trees with more than about 250 genes). I reconciled the resulting gene trees with the appropriate species trees using the NOTUNG software package [13] and varying the bootstrap cut-off parameter; equal weights for gains and losses were used.

### Likelihood analysis of gene families

Using the same 9,920 mammalian and 11,388 *Drosophila *gene families, I used the CAFE software package [35] to estimate the number of gains on each branch of the species trees. The number of gains was calculated by comparing the size of each family between the parent and daughter nodes for each branch; larger daughter-node sizes imply the gain of genes. Gains were then summed across all gene families for each branch. The correlation shown in Figure 7 is with the number of gains inferred using a 90% bootstrap cut-off for the gene tree data. All statistics were calculated in JMP (SAS Institute, Inc. Cary, NC, USA).

## Additional data files

The following additional data are available with the online version of this paper. Additional data file 1 is a figure showing a *Drosophila *species tree. A phylogenetic tree of the 12 species considered in the text is shown. Non-informative branches are marked with an arrow, and the branch preceding the split affected by incomplete lineage-sorting is marked with an asterisk.

## Supplementary Material

Additional data file 1 A phylogenetic tree of the 12 species considered in the text is shown. Non-informative branches are marked with an arrow, and the branch preceding the split affected by incomplete lineage-sorting is marked with an asterisk. Click here for file
