# Supplementary figures and images for: Bias in phylogenetic tree reconciliation methods: implications for vertebrate genome evolution
Source: Genome Biol. 2007 Jul 16;8(7):R141. doi: 10.1186/gb-2007-8-7-r141 (PMC2323230; doi:10.1186/gb-2007-8-7-r141)

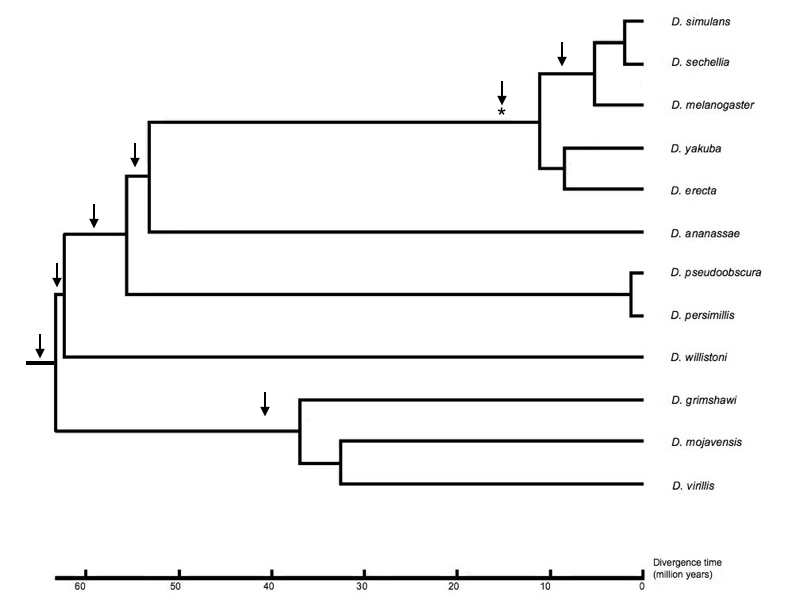

Supplement: Additional data file 1 — A phylogenetic tree of the 12 species considered in the text is shown. Non-informative branches are marked with an arrow, and the branch preceding the split affected by incomplete lineage-sorting is marked with an asterisk. [file gb-2007-8-7-r141-S1.tiff]
